# Salt overload in fructose-fed insulin-resistant rats decreases paraoxonase-1 activity

**DOI:** 10.1186/1743-7075-9-63

**Published:** 2012-06-27

**Authors:** Waleska Cláudia Dornas, Wanderson Geraldo de Lima, Rinaldo Cardoso dos Santos, Melina Oliveira de Souza, Maísa Silva, Mirla Fiuza Diniz, Marcelo Eustáquio Silva

**Affiliations:** 1 Research in Biological Sciences - NUPEB, Federal University of Ouro Preto, Minas Gerais, Brazil; 2 Department of Biological Sciences, Institute of Exact and Biological Sciences, Federal University of Ouro Preto, Minas Gerais, Brazil; 3 Department of Foods, School of Nutrition, University of Ouro Preto, Minas Gerais, Brazil

**Keywords:** Fructose-fed rats, High-salt diet, Paraoxonase, Atherosclerosis

## Abstract

Paraoxonase 1 (PON1) is a HDL-associated esterase/lactonase and its activity is inversely related to the risk of cardiovascular diseases. The aim of the present study was to evaluate the effect of a high-salt diet on serum PON1 activity in fructose-fed insulin-resistant rats. Adult male Fischer rats were initially divided into two groups. Control (CON), which received a normal salt diet and drinking water throughout the study; high fructose (HF), which received a normal salt diet and 20% fructose supplemented drinking water. After 10 weeks, half of the animals from HF group were randomly switched to a high-salt diet and 20% fructose supplemented drinking water (HFS) for more 10 weeks. Serum PON1 activity was determined by synthetic substrate phenyl acetate. HFS rats showed markedly decreased PON1 activity (HFS rats, 44.3 ± 14.4 g/dL versus CON rats, 64.4 ± 13.3 g/dL, *P < 0.05*) as compared to controls. In parallel, the level of oxidative stress, as indicated by thiobarbituric acid reactive substances (TBARS), was increased in HFS rats by 1.2-fold in the liver in relation to controls and was negatively correlated with PON activity. Differential leukocyte counts in blood showed a significant change in lymphocytes and monocytes profile. In conclusion, these results show that PON1 activity is decreased in fructose-fed insulin-resistant rats on a high-salt diet, which may be associated with increased oxidative stress, leading to inflammation.

## Findings

Diabetes is a long-term disorder affecting the inner walls of arteries and is characterized by endothelial dysfunction and oxidative modification of low density lipoprotein (LDL) which may induce atherosclerosis [1]. In contrast, it is supposed that LDL particles can be protected from free radical-induced oxidation by an HDL linked enzyme, paraoxonase 1 (PON1).

Although the precise mechanism of PON1 effect is not yet completely known, it may possess anti-atherogenic and anti-inflammatory properties, resulting from its ability to destroy modified phospholipids and to prevent accumulation of oxidized lipids in lipoproteins [2-5]. In diabetes experimental models, it has been reported that PON1 enzymatic activity is decreased [6,7] and reduced PON1 activity was suggested to play a role in the severity of coronary atherosclerosis [2]. Nonetheless, oxidative stress pathways have also been proposed to act in this process, with increased reactive oxygen species (ROS) production and/or impaired antioxidant defense, which may in turn lead to excessive peroxidation of polyunsaturated fatty acids contained in LDL particles.

Our laboratory has recently reported that a hypercholesterolemic diet in rats cause a significant reduction of PON1 activity, associated with increased oxidative stress [8]. Once rats fed a high dose of fructose are considered a nutritional model for insulin resistance [9-11], the present study investigated the effects of a high-salt diet in fructose-fed insulin-resistant rats on PON1 and its influence in serum and oxidative parameters. In addition the influence of the diets on the inflammatory cells profile was also assessed.

Twenty-nine Fisher male rats, weighing about 300 g, obtained from the School of Nutrition of the Federal University of Ouro Preto, were housed in a temperature and humidity controlled room with a 12:12-h light/dark cycle (lights on at 06.00 AM). Animals were initially divided into two groups. The control group (CON) received the standard AIN93 diet [12] and the high fructose-fed group (HF) was given supplemented fructose as a 20% solution instead of pure water *ad libitum* to induce insulin resistance. After 10 weeks, HF animals were divided into 2 groups: those that continued to receive only 20% fructose in water and those switched to the high fructose regimen + 8% NaCl in the standard AIN93 diet (HFS). After 20 weeks fasting rats were anesthetized and sacrificed. The experiments were approved by the institutional Ethics Committee with protocol number 068/2011 and were performed in accordance with the principles of the Brazilian College of Animal Experimentation [13].

To determine the levels of serum components, blood samples were collected in test tubes and centrifuged. Serum total protein, albumin, total cholesterol, HDL-cholesterol and triglyceride were assayed with colorimetric or enzymatic methods using commercially available kits Labtest (Lagoa Santa, MG, Brazil) # 99, 19, 76, 13 and 87, respectively. The atherogenic index was calculated as follows: total cholesterol - HDL cholesterol/HDL cholesterol.

PON activity was measured by a spectrophotometric assay using phenyl acetate as substrate as described by Beltowski et al. [14]. The enzymatic activity was calculated assuming the molar extinction coefficient of phenylacetate to be 1310 L ^.^ mol^-1^^.^ cm^-1^. The results were expressed in units per milliliter, where 1 U of arylesterase hydrolyzes 1 mmol of phenylacetate per minute. Basal lipid peroxidation status was measured in the liver by TBARS assay [15].

A drop of blood was used to prepare blood smears, which were stained with *Panotic Fast* which use essential components dyes of Romanowsky [16]. Counts of 100 leukocytes were performed and the results were expressed as subtypes identified in polymorphonuclear (PMN), lymphocyte or monocyte cells.

The normality of the sample distribution for each continuous parameter was tested with the Kolmogorov-Smirnov test. The significance of any differences in proportions or medians was tested with Kruskal-Wallis test, and in means analysis of variance (ANOVA) was used, followed by Dunns and Tukey test, respectively. Pearson’s correlation coefficients were used to test the correlation among variables. A p*-*value of less than 0.05 was considered statistically significant.

A high-salt diet in fructose-fed insulin-resistant rats significantly decreased serum levels of total protein, albumin, total cholesterol and HDL-cholesterol after 20 wk of treatment in relation the control group as demonstrated in Table 1. Fructose-fed rats showed significantly higher triglyceride concentrations in both HF and HFS groups (P < 0.05) as compared to control rats. The atherogenic index of each experimental group was also calculated and increased 66% in HFS rats as compared to controls. PON1 activity markedly fell to 68% in those animals, as compared to control and HF rats (P < 0.05 and P < 0.01, respectively) (Table 1).

**Table 1:** Serum total protein, albumin, triglyceride, total cholesterol, HDL-cholesterol, atherogenic index and PON1 activity of experimental rats after 20 weeks of treatment

	**CON**	**HF**	**HFS**
Number of rats/group	9	10	10
Total protein (g/dL)	7,0 ± 2,0^a^	6,3 ± 1,8^a^	4,9 ± 1,5^b^
Albumin (g/dL)	2,8 ± 0,2^a^	2,6 ± 0,3^a^	2,1 ± 0,2^b^
Triglyceride (mg/dL)	126,1 ± 30,8^b^	183,5 ± 58,7^a^	197,1 ± 55,9^a^
Total cholesterol (mg/dL)	97,9 ± 20,9^a^	98,0 ± 13,3^a^	69,3 ± 9,0^b^
HDL-cholesterol (mg/dL)	66,4 ± 10,6^a^	65,8 ± 9,4^a^	43,6 ± 12,17^b^
Atherogenic index	0,39 ± 0,12^b^	0,47 ± 0,11^b^	0,65 ± 0,26^a^
PON1 (g/dL)	64,45 ± 13,36^a^	65,44 ± 17,29^a^	44,3 ± 14,43^b^

Values (mean ± SD) were obtained from groups CON (AIN diet and water for 20 weeks); HF (AIN diet + 20% fructose solution in water for 20 weeks) and HFS (8% NaCl in AIN93 diet + 20% fructose solution in water for 10 weeks after previous treatment for a period of 10 weeks with HF). Different superscript letters within the same row indicate significant difference at P < 0.05 by one-way ANOVA followed by Tukey’s test. High-density lipoprotein (HDL); paraoxonase1 activity (PON1).

**Figure 1:**
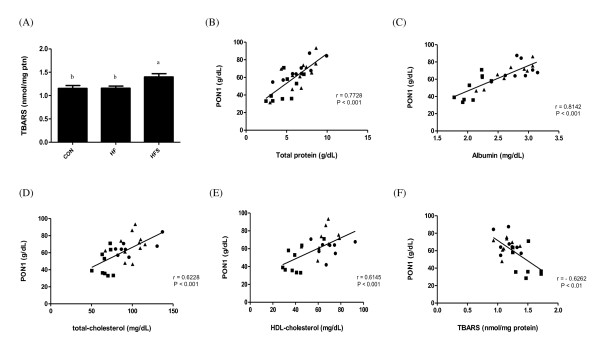
**(A) Liver TBARS levels; scatter plots illustrating the association between (B) PON1 and total protein and (C) albumin and (D) Total cholesterol and (E) HDL-cholesterol and (F) TBARS in experimental groups.** Control (*dark filled circle*); high-fructose (*dark filled triangle*) and high-fructose + salt (*dark filled square*) rats. High-density lipoprotein (HDL); paraoxonase1 activity (PON1).

Increased oxidative stress was noted in the liver obtained from HFS rats, since TBARS levels were raised by 1.2 times (P < 0.05) as compared to controls (Figure 1A). When data from all experimental groups were analyzed together, the effects of all treatments on serum PON1 activity were positively correlated with total protein (r = 0.7728, P < 0.001) (Figure 1B), albumin (r = 0.8142, P < 0.001) (Figure 1C), total cholesterol (r = 0.6228, P < 0.001) (Figure 1D) and HDL-cholesterol (r = 0.6145, P < 0.001) (Figure 1E). In addition, a negative correlation was found between serum PON1 activity and liver TBARS levels (r = − 0.6262, P < 0.01) (Figure 1F).

Differential leukocyte counts in the total blood smear in insulin-resistant rats treated or not with the high-salt diet showed a significant decrease in the lymphocyte profile in relation to the control group, but the percentage of polymorphonuclear cells (PMNs) remained unchanged. Salt treatment caused a significant increase in the monocyte profile in HF and HFS rats as compared to the CON rats (Table 2).

**Table 2:** Polymorphonuclear cells, lymphocytes and monocytes count of experimental rats after 20 weeks of treatment

	**CON**	**HF**	**HFS**
PMN cells (%)	5-15	3-27	7-23
Lymphocytes (%)	19-58	4-33^**^	7-20^***^
Monocytes (%)	27-76	36-85^**^	40-83^*^

Values were obtained from groups CON (AIN diet and water for 20 weeks); HF (AIN diet + 20% fructose solution in water for 20 weeks), and HFS (8% high salt in AIN93 diet + 20% fructose solution in water for 10 weeks after previous treatment for a period of 10 weeks with HF). Kruskall Wallis test followed by Dunns test. Values indicate lowest and highest percentage of cells counted respectively. ^*^P < 0.05; ^**^P < 0.01; ^***^P < 0.001. Polymorphonuclear (PMN) cells.

In this study we observed a decrease in serum PON1 activity in insulin-resistant rats on a high-salt diet and an association among PON1, oxidative stress and inflammation, which can occur during the development of atherosclerosis.

HDL is the most powerful independent negative predictor of cardiovascular events. The protective effects of HDL have been first attributed to its capacity to promote cellular cholesterol efflux from peripheral cells and deliver it to the liver for excretion, a process known as reverse cholesterol transport [1]. Furthermore HDL has also attracted particular attention because of its antioxidative potential. Interestingly this work sustains the relationship between low paraoxonase activity and diseases with low antioxidant defense and excessive lipid peroxidation since paraoxonase may act protectively in atherogenesis by hydrolyzing some products of lipid peroxidation and consequently by limiting LDL oxidation and foam cell formation [17].

In our model, serum albumin and protein levels are reduced probably due to protein synthesis disruption in the liver as observed by Shinn and Moon [18]. The damaged hepatocytes are potent sources of reactive oxygen intermediates and oxidative stress is important in many types of liver injury. Lipid peroxidation, which was found increased in this study, could change the properties of biological membranes, resulting in severe cell damage and play a significant role in the mechanisms related to the alterations observed by us, as it has been previously documented [19].

In rats, unlike in humans and rabbits, plasma triglycerides and cholesterol are transported predominantly by HDL [14] and fructose per se, after having reached the liver, may contribute to the increase in serum triglyceride as demonstrated in this study and by others authors [9,20,21]. We showed that low PON1 activity is associated with decreased HDL levels and this contributes to the hypothesis that PON1 is involved in the preservation of HDL. Since PON1 exerts a protective effect against oxidative stress, it is logical to find an association between this enzyme and liver impairment. PON1 activity decreased while lipid peroxidation increased in HFS rats, suggesting that PON1 activity may be involved in the defense against free radical production in liver organelles. Moreover, the atherogenic index of these animals indicated them to be more susceptible to the development of atherosclerosis. However the results of this study showed that PON1 activity towards synthetic substrates reflects its relationship to oxidative stress by means of the marker used but this hypothesis needs support by means of further investigation.

A previous study has shown that PON1 protects against atherosclerosis, by its ability to reduce macrophage foam cell formation, via reducing oxidative stress and stimulation of cholesterol efflux from macrophages [22]. The present study proposes that oxidatively modified LDL impair endothelial function, stimulate inflammatory response of monocytes/macrophages, activate migration and proliferation of vascular smooth muscle cells and induce immune response, as indicated by the increase of monocytes in HF and HFS groups. Therefore, inactivation of PON1 in HFS rats could reduce the ability of HDL to inhibit LDL modification and also decrease the ability of HDL to inhibit monocytic-endothelial interactions. Both conditions appear to be important in the inflammatory response in arterial wall cells, as demonstrated by the change in lymphocyte profile in relation to the control group, since this is a common finding during the systemic inflammatory response. In addition, clinical and animal studies suggest that low lymphocyte count plays a putative role in chronic inflammation [23].

Our dietetic model was chosen due its relevance to human nutrition because it mimics a common Western diet, with high consumption of sugar drinks and salty foods and this study is the first one to show an association between paraoxonase status and salt overload in insulin-resistant rats. This supports the relationship between low PON1 activity and diseases with low antioxidant defense and excessive lipid peroxidation. Paraoxonase may perform protectively in atherogenesis by hydrolyzing some products of lipid peroxidation and consequently by limiting LDL oxidation and foam cell formation [24]. This allows us to hypothesize that paraoxonase activity may lead to failure in the protection of LDL oxidation in insulin-resistant subjects on a high-salt diet. Thus the lack of prevention of LDL oxidation by HDL could be explained by the quantitative alteration of HDL, since PON1 generally seems to contribute to the antioxidant properties of HDL.

In conclusion, the present study indicates that a high-salt diet in fructose-fed rats leads to lowered serum PON1 activity, with deleterious effects on the antioxidant defense system. Furthermore, we suggest that a change in blood cells profile would reflect the oxidative stress detected. These findings we believe to be relevant in the field of human nutrition.

## Abbreviations

HDL, High-density lipoprotein; LDL, Low-density lipoprotein; PMNs, Polymorphonuclear; PON, Paraoxonase1; ROS, Reactive oxygen species; TBARS, Thiobarbituric acid reactive substances.

## Competing interests

The authors declare that they have no competing interests.

## Author’s contributions

WCD and MES participated in the design of the study, interpretation of data and edited the manuscript. WCD, MOS, MS, MFD collected the data. RCS made substantial contributions in revising it critically for intellectual content. WGL contributed to the discussion of data and statistical analysis. MES coordinated the study. All authors read and approved the final version of the manuscript.
